# Interruption of Transmission of *Onchocerca volvulus* in the Southern Chiapas Focus, México

**DOI:** 10.1371/journal.pntd.0002133

**Published:** 2013-03-28

**Authors:** Mario A. Rodríguez-Pérez, Alfredo Domínguez-Vázquez, Thomas R. Unnasch, Hassan K. Hassan, Juan I. Arredondo-Jiménez, María Eugenia Orozco-Algarra, Kristel B. Rodríguez-Morales, Isabel C. Rodríguez-Luna, Francisco Gibert Prado-Velasco

**Affiliations:** 1 Centro de Biotecnología Genómica, Instituto Politécnico Nacional, Reynosa, Tamaulipas, México; 2 Onchocerciasis Elimination Program for the Americas, Guatemala City, Guatemala; 3 Global Health Infectious Disease Research Program, Department of Global Health, College of Public Health, University of South Florida, Tampa, Florida, United States of America; 4 Dirección de Enfermedades Transmitidas por Vector, Centro Nacional de Vigilancia Epidemiológica y Control de Enfermedades, Secretaria de Salud, México D.F., Mexico; 5 Programa para la Eliminación de la Oncocercosis en Chiapas, Departamento de Prevención y Control de Enfermedades Transmitidas por Vector, Tuxtla Gutiérrez, Chiapas, México; Michigan State University, United States of America

## Abstract

**Background:**

The Southern Chiapas focus of onchocerciasis in Southern Mexico represents one of the major onchocerciasis foci in Latin America. All 559 endemic communities of this focus have undergone semi-annual mass treatment with ivermectin since 1998. In 50 communities of this focus, ivermectin frequency shifted from twice to four times a year in 2003; an additional 113 communities were added to the quarterly treatment regimen in 2009 to achieve a rapid suppression of transmission.

**Methodology/Principal findings:**

In-depth epidemiologic and entomologic assessments were performed in six sentinel communities (which had undergone 2 rounds of ivermectin treatment per year) and three extra-sentinel communities (which had undergone 4 rounds of ivermectin treatment per year). None of the 67,924 *Simulium ochraceum* s.l. collected from this focus during the dry season of 2011 were found to contain parasite DNA when tested by polymerase chain reaction-enzyme-linked immunosorbent assay (PCR-ELISA), resulting in an upper bound of the 95% confidence interval (95%-ULCI) of the infective rate in the vectors of 0.06/2,000 flies examined. Serological assays testing for *Onchocerca volvulus* exposure conducted on 4,230 children 5 years of age and under (of a total population of 10,280 in this age group) revealed that 2/4,230 individuals were exposed to *O. volvulus* (0.05%; one sided 95% confidence interval = 0.08%).

**Conclusions/Significance:**

The in-depth epidemiological and entomological findings from the Southern Chiapas focus meet the criteria for interruption of transmission developed by the international community.

## Author Summary

The absence of infective larvae of *Onchocerca volvulus* in the black fly vector of this parasite and reduction of exposure to and new infections with *O. volvulus* are the criteria currently used to certify focal interruption of parasite transmission. In the present study, we report entomological and epidemiological assessments in the Southern Chiapas focus of Mexico that together indicates that transmission of *O. volvulus* has been interrupted in this focus. None of the *Simulium ochraceum* s.l. vector black flies collected from sentinel and extra-sentinel communities in this focus during the 2011 transmission season was found to contain parasite DNA when tested by PCR-ELISA, suggesting vector parasite contact was nearly nonexistent. In addition, there was a minimal exposure to the parasite in children 5 years of age and under, as measured by circulating antibody to a parasite-specific antigen. The Southern Chiapas focus was the major focus in Mexico and one of the largest in Latin America, with well-documented history of active transmission prior to the commencement of Mectizan mass distribution. This study demonstrates the interruption of transmission in geographically large focus in Latin America with a historically high intensity of transmission.

## Introduction

Human onchocerciasis is caused by the filarial parasitic nematode *Onchocerca volvulus*, which in Latin America is transmitted by new world black flies (*Simulium* spp.) in six countries (Brazil, Colombia, Ecuador, Venezuela, Guatemala, and Mexico), where 525,543 individuals are at risk [1]. In Mexico, onchocerciasis was endemic in three distinct foci (Southern Chiapas, Northern Chiapas, and Oaxaca). The Southern Chiapas focus was the major focus in Mexico given its large size (12,579.70 km^2^) and well-documented history of intense transmission. The Southern Chiapas focus contains 559 affected communities, 39 of which were hyperendemic for onchocerciasis before control efforts began, while 209 of the communities were mesoendemic, and 311 were hypoendemic. The population at risk in the Southern Chiapas focus (114,024 individuals) comprised about 21% of the total at-risk population in the Americas.

Since the 1990s, onchocerciasis control in Mexico has relied on the mass distribution of Mectizan (ivermectin) to the at-risk communities. Annual mass ivermectin distribution treating to all eligible residents from the at-risk communities began in 1994. In 1998 the strategy was modified to provide mass treatments every 6 months. In 50 communities from the Southern Chiapas focus, the ivermectin distribution frequency again shifted in 2003, from twice to four times a year. An additional 113 communities were added to the quarterly treatment regimen in 2009.

The goal of the Onchocerciasis Elimination Program for the Americas [1] is to eliminate new ocular morbidity caused by infection with *O. volvulus* and eventually eliminate transmission of the parasite in all 13 foci in Latin America. The World Health Organization (WHO) [2] and OEPA [3], [4] have established a series of epidemiologic and entomologic criteria to be achieved to declare onchocerciasis eliminated. These include a reduction of new infections to an incidence rate of less than one new case per 1,000 individuals (<0.1%) [2], [3] and an absence, or near absence, of infective-stage larvae of *O. volvulus* in the vector population (i.e., a rate of less than one infective fly per 1,000 parous flies). Practically, because polymerase chain reaction (PCR) using *O. volvulus*-specific DNA probes are generally applied to examine pools of flies, parity cannot be easily determined, so the threshold used is less than one infective fly per 2,000 flies tested (assuming a 50% parity rate) [2], [3], [4]. The data presented here report results of in-depth epidemiological and entomological studies conducted in 2007 through 2011 which, when taken together suggest that, based upon these criteria, that *O. volvulus* transmission has been interrupted in the Southern Chiapas focus.

## Materials and Methods

### Selection of communities and study area

**Figure 1:**
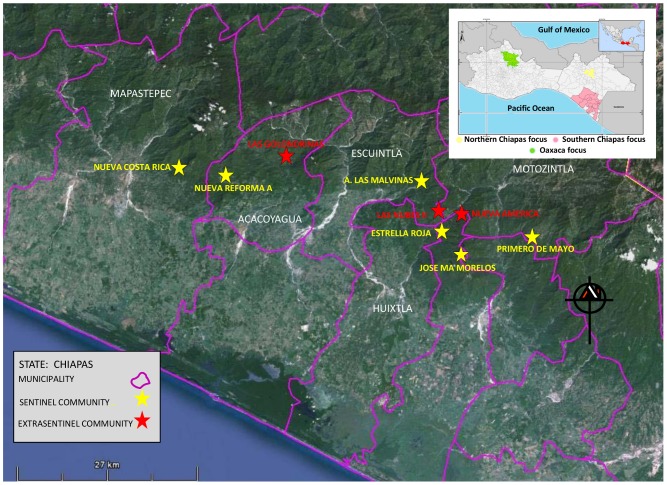
Map of the Southern Chiapas focus, México. The map of the Southern Chiapas focus shows the six sentinel (marked in yellow) and three extra-sentinel communities (marked in red) and the main rivers and tributaries that might serve as a source of black fly breeding. Map (right) of the Southern Mexico states showing the three onchocerciasis endemic foci.

In 1995, local health authorities selected six sentinel communities of the Southern Chiapas focus (Figure 1). These included Ampliación Las Malvinas (Escuintla municipality; 92°28′24″W, 15°20′36″N, elevation 1,000 masl), Estrella Roja and José María Morelos (Huixtla municipality; 92°28′48″W, 15°16′11″N and 92°27′35″W, 15°13′48″N, elevation 660 and 1400 masl, respectively), Nueva Costa Rica (Mapastepec municipality; 92°48′46″W, 15°28′01″N, elevation 600 masl), Nueva Reforma Agraria (Acacoyagua municipality; 92°45′04″W, 15°26′04″N, elevation 500 masl), and Primero de Mayo (Motozintla municipality; 15°14′31″W, 92°18′04″N, elevation 1,300 masl). Following the addition of 50 communities to the quarterly treatment in 2003, two additional extra-sentinel communities were selected. These included Las Golondrinas (Acacoyagua municipality; 92°39′17″W, 15°26′06″N, elevation 920 masl) and Las Nubes II (Escuintla municipality; 92°31′49″W, 15°23′09″N, elevation 1,200 masl). In 2008, an additional extra-sentinel community was added; Nueva América (Huixtla municipality; 92°26′40″W, 15°17′00″N, elevation 880 masl) (Figure 1).

These communities were the ones evaluated in this study. These communities have to varying extents been included in previous in-depth epidemiological evaluations (EEP) which have been carried out in the Southern Chiapas focus (Table 1). The EEPs in these communities have permitted the evaluation of the impact of mass treatment with ivermectin on transmission and have provided the baseline data employed to demonstrate that transmission has been interrupted.

**Table 1:** In-depth epidemiologic assessments in sentinel and extra-sentinel communities of the Southern Chiapas focus.

Surveys	1995	1998	2000	2001	2004	2006	2008	2010	2011
**Ophthalmological**	X		X		X	X	X		
**Parasitological**	X	X	X		X	X	X		
**Entomological**				X	X	X	X	X	X
**Serological**					X	X	X	X	

Apart from the sentinel and extra-sentinel communities, a large-scale serological study was performed during 2010 on 4,230 children resident in 110 extra-sentinel communities with high historical endemicity. This sampled group represented 41% of all children five and under who were residents in these historically highly endemic communities.

**Figure 2:**
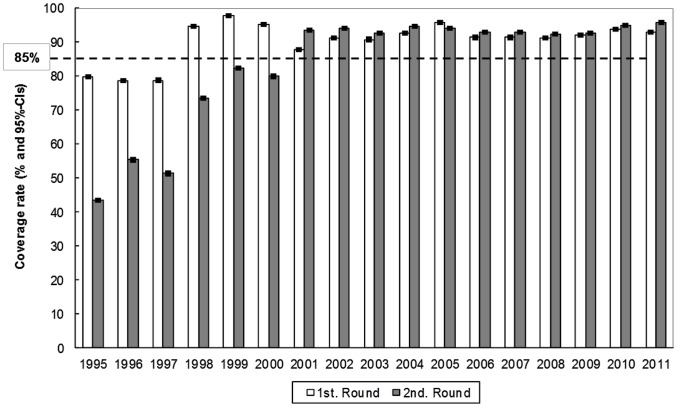
Coverage rate with ivermectin of the eligible population of the Southern Chiapas focus. The coverage rate, expressed in percent and the 95%-confidence intervals, CIs, surrounding point estimate, with ivermectin of the eligible population of the Southern Chiapas focus, 1995–2011. The line at 85% indicates the coverage needed in a sustained fashion to interrupt transmission.

In the Southern Chiapas focus, ivermectin distribution was commenced in 1990, but was initially only offered to registered onchocerciasis “clinical cases”. In 1994, every eligible resident in the hyperendemic and mesoendemic communities was treated, but only 25% of the eligible residents from hypo-endemic communities were treated. From 1995 to 1997, the 40% of the eligible population in hypo-endemic communities was treated. Beginning in 1998, the entire eligible populations in all communities, regardless of their level of endemicity, were provided with semi-annual ivermectin treatments. In 50 communities of this focus, treatments were increased from twice to four times per year in 2003; the quarterly treatment regimen was extended to 113 communities in 2009. A total of 22 consecutive treatment rounds reaching >85% of the eligible population have been provided in the Southern Chiapas focus over the past 11 years. Ivermectin coverage of the eligible population has remained at a level greater than 85% every year from 1998 through 2011 (Figure 2).

### Entomological survey

Black flies (*S. ochraceum* s.l) were collected in the sentinel and extra-sentinel communities using standardized procedures [5], [6], [7], [8], [9] during the peak *O. volvulus* transmission season, lasting from December to March. Collections were carried out during the first 50 minutes of each hour, beginning at 11:00 AM and ending at 4:50 PM [10].

Black flies were collected before they began feeding. The landing rate measured from the collections was taken as an estimate of the biting rate, although this probably overestimated the biting rate, because a proportion of the landing flies in a natural setting do not successfully obtain a blood meal. Thus, the transmission potential calculations provided below are likely to be overestimated by a factor proportional to the number of flies that land but do not bite.

Flies were combined into pools containing a maximum of 50 individuals per pool and the heads and bodies separated as previously described [5]. The separated head and body pools were tested for *O. volvulus* parasites by using a PCR assay specific for *O. volvulus*. Details of protocols for genomic DNA purification, primer sequences, PCR conditions, and detection of PCR products by enzyme-linked immunosorbent assay (ELISA) have been published elsewhere [5], [11]. In brief, DNA extractions were carried out in sets of 20 samples each, with each set containing 18 fly pools and two sham extractions which served as contamination controls for the DNA extraction process. All PCRs were carried out in sets of 84 samples, in rows B–H of a PCR microtiter plate. Row A was reserved for 10 PCR-negative controls and two positive controls. One positive control contained the minimal amount of positive control DNA consistently detected by the PCR amplification conditions, as determined by an initial titration study. This control was carried out to ensure that all of the reactions were operating at peak efficiency. The second positive control contained the same minimal amount of positive control DNA mixed with 2.5 µL of a DNA preparation from a fly pool that tested negative in a prior set of reactions. This control ensured that no inhibitors were present in the fly DNA preparations. Initial screenings focused on pools of bodies, as previous studies have shown that infection rates in bodies provide a more sensitive indicator of parasite-vector contact than testing heads [5], [11]. All head pools were screened from any community found to have evidence of any vector-parasite contact based upon the body pool screens, providing an estimate of the prevalence of flies carrying infective larvae. PoolScreen v2.0 was used to estimate the prevalence of infected flies in the community and the associated 95% confidence intervals (CIs) [12].

Seasonal transmission potentials [STP] for each sentinel and extra-sentinel village were calculated as the product of the seasonal biting rate, the proportion of flies carrying L3 larvae in the transmission season (from December through March), and the average number of L3 larvae in each infective fly. As previously discussed, after multiple rounds of Mectizan treatment, the number of infective larvae present in each infective fly was assumed to be one [8], [9], [11].

The seasonal biting rate was calculated as the product of the geometric mean [13] of the number of flies collected per person per day and the total number of days in the transmission season, which included the months of December through March. The daily biting rate and the seasonal biting rate were estimated as previously described [8], [9], [14]. Because *S. ochraceum* s.l. females were not collected throughout the year, it was not possible to precisely calculate the annual transmission potential (ATP). However, given the paucity of vector black flies present outside the normal transmission season, the transmission potential outside of the peak transmission period is probably zero or near zero. The STP (transmission occurring during the peak transmission season of December through March) thus likely represented a fairly accurate estimate of ATP.

### Serological survey

The prevalence of IgG4 antibodies to Ov16 [15], [16], a recombinant antigen of *O. volvulus*, was determined from two populations of children in the Southern Chiapas focus: residents in the sentinel and extra-sentinel villages, and school children selected from the overall population sample in the focus. In 110 communities (including all previously mesoendemic and hypoendemic communities in the focus), blood spots were collected from 4,313 children 5 years of age and under. Sera from 4,230 children were screened for Ov16 antibodies.

Blood spots were collected by finger prick from each individual enrolled in the study, dried in the field, transported to the laboratory at 4°C, and kept refrigerated in sealed bags containing silica gel at −20°C until use, which occurred within a month of collection. Two 6-mm punches of blood saturated filter paper were placed in a phosphate-buffered saline-Tween (PBS-T) 0.05% and bovine serum albumin (BSA) 5% buffer and eluted overnight at 4°C. The elution was then run in duplicate in a standard ELISA [5], to detect IgG4 antibodies against the OV-16 recombinant antigen. A standard curve was used on each plate to identify positive samples and permit comparisons between plates and over days. The cut-off value was determined after analyzing OV-16 negative and OV-16 positive samples (from 10 parasitologically confirmed *O. volvulus* positive individuals). The cutoff was chosen as 40 arbitrary units by identifying the value that optimized both sensitivity and specificity. Any positive results were repeated before being reported as positive.

### Ophthalmological survey

Ocular examinations were carried out by an ophthalmologist experienced in onchocerciasis ocular evaluations for OEPA. The examinations were done using a Topcon Optical SL-3D slit lamp (Kogaku Kikai KK, Tokyo, Japan). Exams focused on finding *O. volvulus* microfilariae in the cornea (MFC) and/or the anterior chamber of the eye (MFAC). Before the exam the patients kept a “head down position” (forehead in the lap) for 5 minutes to allow MFC and/or MFAC to settle in a visible position. In 2007 and 2008, a population of 1,418 and 326 residents, representing about 72% and 74% of the total population in the six sentinel and the three extra-sentinel communities were examined.

### Parasitological survey

Nodulectomy campaigns have been undertaken in Mexico since 1932. Individuals that undergo nodulectomy received beneficial effects as their mf loads and skin pathology were reduced. Hence, nodulectomy was considered a routine treatment provided by the Mexican elimination program. The number of individuals with nodules of the Southern Chiapas focus was registered from 1995 through 2010. In parallel, the number of new clinical cases (i.e., individuals diagnosed positive for nodules or skin microfilariae for the first time) were also registered from 1989 through 2010. The skin biopsies were taken from each patient using a 1.5- to 2.0-mm corneoscleral biopsy punch. Skin biopsies were incubated overnight in buffered saline, and emerging microfilariae were visualized and counted using an inverted microscope.

### Ethics statement

The procedures were reviewed and approved by the Presidents of the Ethical and Bio-security Committees of the National Institute of Public Health (Cuernavaca, México) and the Health Secretariat of México (The city of México, D.F.). These are the equivalents of the Institutional Review Boards for Human Subject Research in the USA. Community meetings were held in all selected villages within the focus to explain the research procedures, and the right of each individual to decide whether or not to participate was explained. The individuals were also informed that they would be provided with the results of the tests upon request. Before each examination, each adult who had voluntarily come to the examination point and agreed to participate were provided with a capsule summary of the project and process and oral consent was obtained. Parents or guardians provided oral consent on behalf of all child participants. The Ethical Committee of the Health Secretariat of México approved the use of oral consent, given that the studies were conducted as part of the national onchocerciasis surveillance program and were therefore part of a routine public health monitoring program conducted by the Mexican government.

Community leaders were also consulted and approved the use of the selected locations in the village and river banks as vector catching points.

### Statistical analysis

PoolScreen v2.0 was used to calculate a prevalence of infection and associated 95% CIs in the vector populations. The prevalence of infective flies was then combined with estimates of the biting rate (calculated from the fly collection data as described previously) to calculate an estimated STP. The proportion of individuals with mf in the cornea and/or anterior chamber of the eye was calculated as the number of positive individuals divided by the total number examined, and expressed as a percentage. The associated 95% exact CIs of the proportion of individuals harboring Ov16 antibodies from the large-scale serology study were determined using the method of Thompson [17]. The method of Miettinen (1970), as described in Armitage and Berry [18] was used to estimate the 95% exact CIs surrounding the point prevalence of Ov16 antibodies, MFC and MFAC of the sentinel and extra-sentinel communities.

## Results

### Entomological survey

In 2008, a total of 103,610 *S. ochraceum* s.l. (68,616 in the sentinel and 34,994 n the extra-sentinel communities) were subjected to analysis by PCR. The number of flies tested in each community was sufficient to comply with the WHO guideline of having at least 10,000 flies tested from each community. The results are summarized in Table 2. The point estimate of the prevalence of infective flies and the associated 95%-upper limit confidence interval (ULCI) in all communities were both well below the threshold of 1/2,000 (maximum 0.07/2,000 and 0.1/2,000 flies examined, respectively).

**Table 2:** Entomological parameters in the focus of Southern Chiapas, México.

Community (year of entomologic study)	Seasonal biting rate	Prevalence of Infective flies/2,000*	Seasonal transmission potential*
**Sentinel communities (2008)**			
Primero de Mayo	86,009	**0** (0.3)	**0** (12.9)
Ampliación Malvinas	14,111	**0** (0.5)	**0** (3.7)
Estrella Roja	14,628	**0** (0.4)	**0** (2.9)
José María Morelos	102,020	**0** (0.4)	**0** (18.4)
Nueva Costa Rica	38,753	**0** (0.4)	**0** (7.8)
Nueva Reforma Agraria	35,690	**0** (0.4)	**0** (7.9)
**Extra-sentinel communities (2008)**			
Las Golondrinas	81,701	**0** (0.3)	**0** (12.3)
Las Nubes II	83,237	**0** (0.3)	**0** (12.5)
Nueva América	57,246	**0** (0.3)	**0** (9.7)
**Other communities (2009–2010)**			
Brasil	60,183	**0.3** (0–0.7)	**7.5** (0–21.1)
Mexiquito	83,038	**0.4** (0.2–1.0)	**16.6** (8.3–42.3)
Coronado Santa Rita	51,720	**0** (0.4)	**0** (10.3)
Loma Bonita	14,278	**0** (0.9)	**0** (6.4)
Montawa	9,390	**0** (1.3)	**0** (6.1)
La Granja	3,142	**0** (4.1)	**0** (6.4)
La Soledad	10,311	**0** (1.1)	**0** (5.7)
**Sentinel communities (2010–2011)**			
Estrella Roja	7,530	**0** (0.6)	**0** (2.3)
José María Morelos	85,657	**0** (0.3)	**0** (12.8)
**Other communities and Coffee fincas (2010–2011)**			
Brasil	58,732	**0** (0.3)	**0** (8.8)
Mexiquito	81,338	**0** (0.3)	**0** (12.2)
Finca La Victoria	50,282	**0** (0.3)	**0** (7.5)
Finca Santa Amalia	36,250	**0** (0.4)	**0** (7.3)

The seasonal *S. ochraceum* s.l. biting rate is the number of bites per person per season. Prevalence of infective flies is expressed as rate per 2,000 flies examined and seasonal transmission potential is the third-stage larvae per person per season. These entomological parameters were estimated during 2008 through 2011 in the sentinel, extra-sentinel and other communities in the focus of Southern Chiapas.

* Value represents point estimate and value in parentheses represents 95%-confidence intervals, CIs (95% upper limit CI when zero) surrounding point estimate.

In 2011, follow-up entomologic studies were carried out (Table 2). Two sentinel communities (José María Morelos and Estrella Roja) were included in the follow up study. A total of 67,924 flies were collected and tested from these two communities, and all body pools were negative for *O. volvulus* DNA. Similarly, flies were collected in two communities (Brasil and Mexiquito) where evidence of *O. volvulus* DNA in the vector was seen in entomological studies carried out in 2009–2010. None of the pools collected in 2011 were positive (Table 2). Finally, flies were collected and tested from two coffee fincas (La Victoria and Santa Amalia); these were also all found to be negative for parasite DNA (Table 2). Taken together, these data suggested no parasite-vector contact was occurring throughout the entire focus. The 95%-ULCI surrounding point prevalence of infective flies in all areas was well below the threshold of 1/2,000 (maximum 0.06/2,000 flies examined). The upper bound of the 95% CI for the STPs ranged from 0.0 to 1.0 L3 per person per season.

### Serological survey

The results of the serological surveys are shown in Table 3. In 2010, only two children 5 and under from 110 communities within the focus were positive for Ov16 IgG4 antibodies out of a total of 4,230 children examined (point prevalence = 0.05%). The upper bound of the one sided 95% confidence interval (calculated taking into account that a large proportion of a finite population had been sampled) was 0.08%.

**Table 3:** Prevalence of IgG4 antibodies to Ov16 in the focus of Southern Chiapas, México.

**Sentinel communities (2008)**						
Primero de Mayo	Ampliación Malvinas	Estrella Roja	José María Morelos	Nueva Costa Rica	Nueva Reforma Agraria	**Total**
0/37 (0.0%)	1/71 (1.4%)	2/119 (1.7%)	1/87 (1.1%)	0/227 (0.0%)	0/135 (0.0%)	**4/676 (0.6%)**
**Extra-sentinel communities (2008)**						
Las Golondrinas	Las Nubes II	Nueva América				
0/100 (0.0%)	0/38 (0.0%)	3/133 (2.3%)				**3/271 (1.1%)**
**Other communities (2009)**						
Ranchería Las Marías	Barrio El Retiro	La Paz de Sabines	Col Libertad el Pajal	Montecristo de Guerrero	S. Antonio Miramar	
0/50 (0.0%)	0/48 (0.0%)	0/106 (0.0%)	0/128 (0.0%)	0/452 (0.0%)	0/20 (0.0%)	
El Vergel	Plan de Ayala					
1*/122 (0.8%)	0/120 (0.0%)					**1** * **/1,046 (0.09%)**
**Coffee fincas (2009)**						
Finca Santa Amalia	Finca Victoria	Finca Santa Fé				
2/224 (0.9%)	1/456 (0.2%)	0/143 (0.0%)				**3/823 (0.4%)**
**110 communities within 4 districts (2010)**						
Comitán	Villaflores	Tapachula	Tonalá			
0/918 (0.0%)	0/1,338 (0.0%)	2/1,869 (0.1%)	0/105 (0.0%)			**2/4,230 (0.05**%)

Prevalence of IgG4 antibodies to Ov16 in children 10 years of age and under from sentinel, extra-sentinel, and eight other communities, in migrant workers >15 years of age from three coffee fincas and in children 5 years of age and under from 4 districts in the focus of Southern Chiapas, México.

* Skin biopsies were taken and tested by PCR; they contained no parasite DNA (unpublished data).

### Ophthalmological survey

No microfilariae in the cornea were observed; two individuals harboring microfilariae in the anterior chamber of the eye were identified in 1,744 residents examined during 2007 and 2008 in the sentinel and extra-sentinel communities, (point prevalence 0.07%; 95% CI = 0.001–0.4%; Table 4).

**Table 4:** Total population and individuals examined by parasitology and ophthalmology in Southern Chiapas focus.

Community	Total population/Examined by parasitology/Examined by ophthalmology					
	1995	1998	2000	2004	2006	2008
**Sentinel communities**						
Primero de Mayo	213/175/80	226/118/ND	211/108/87	178/150/104	153/ND/103	137/126/ND
Ampliación Malvinas	184/120/85	204/139/ND	231/135/28	214/160/108	206/ND/96	227/199/ND
Estrella Roja	248/179/100	306/40/ND	351/146/ND	330/263/170	304/ND/234	305/264/ND
José María Morelos	358/158/75	358/181/ND	406/95/163	360/228/181	329/ND/221	322/305/ND
Nueva Costa Rica	542/520/276	722/305/ND	759/409/396	667/635/433	663/ND/500	729/653/ND
Nueva Reforma Agraria	305/233/135	372/254/ND	378/297/188	343/322/203	318/ND/254	385/340/ND
**Extra-sentinel communities**						
Las Golondrinas				303/285/186	323/317	333/325/273
Las Nubes II				137/111/73	118/105	121/113/95
Nueva América						439/386/326

The number of communities, number of people in total, and number of people examined by parasitology and ophthalmology in the sentinel and extra-sentinel communities of the Southern Chiapas focus, México.

ND = No data.

### Parasitological survey

**Figure 3:**
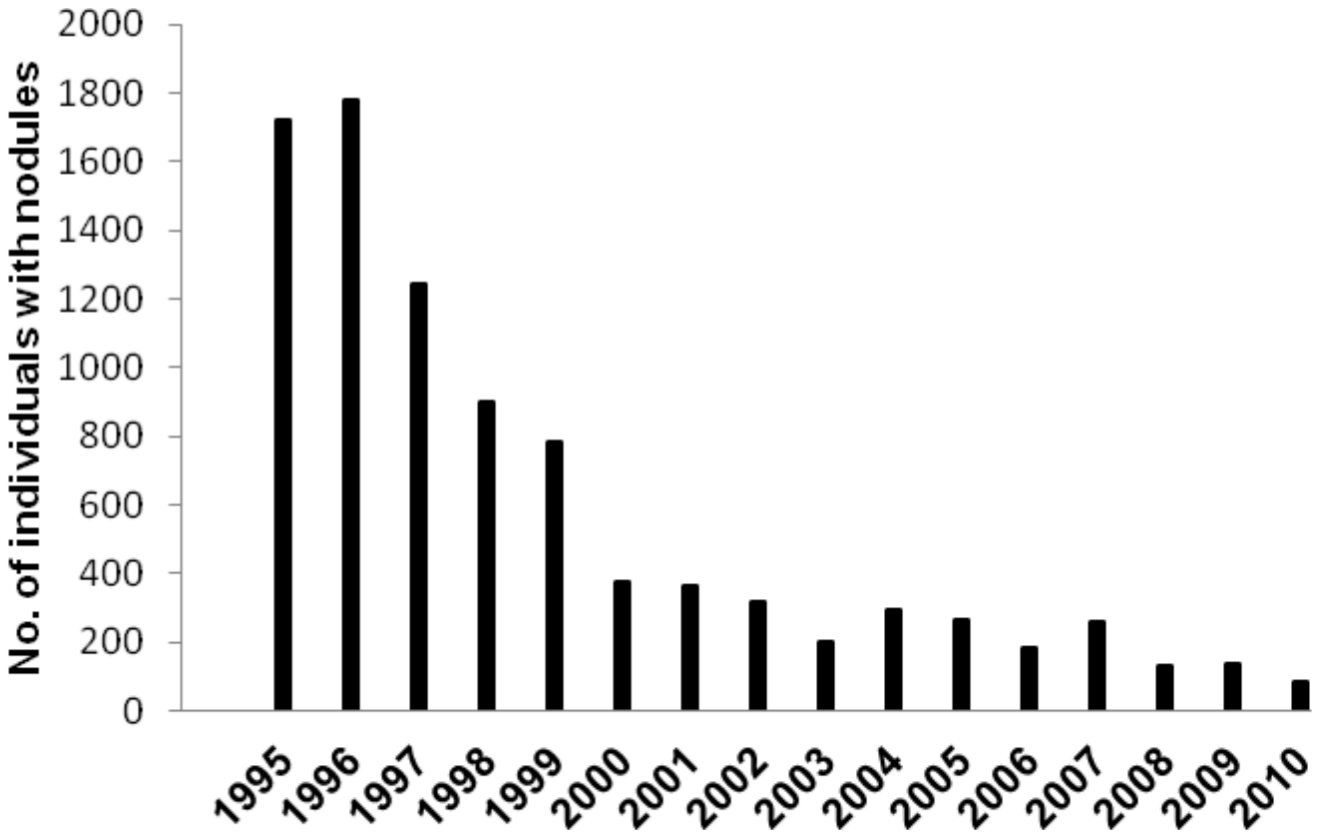
Number of individuals with nodules in the Southern Chiapas focus, Mexico, 1995–2010.

**Figure 4:**
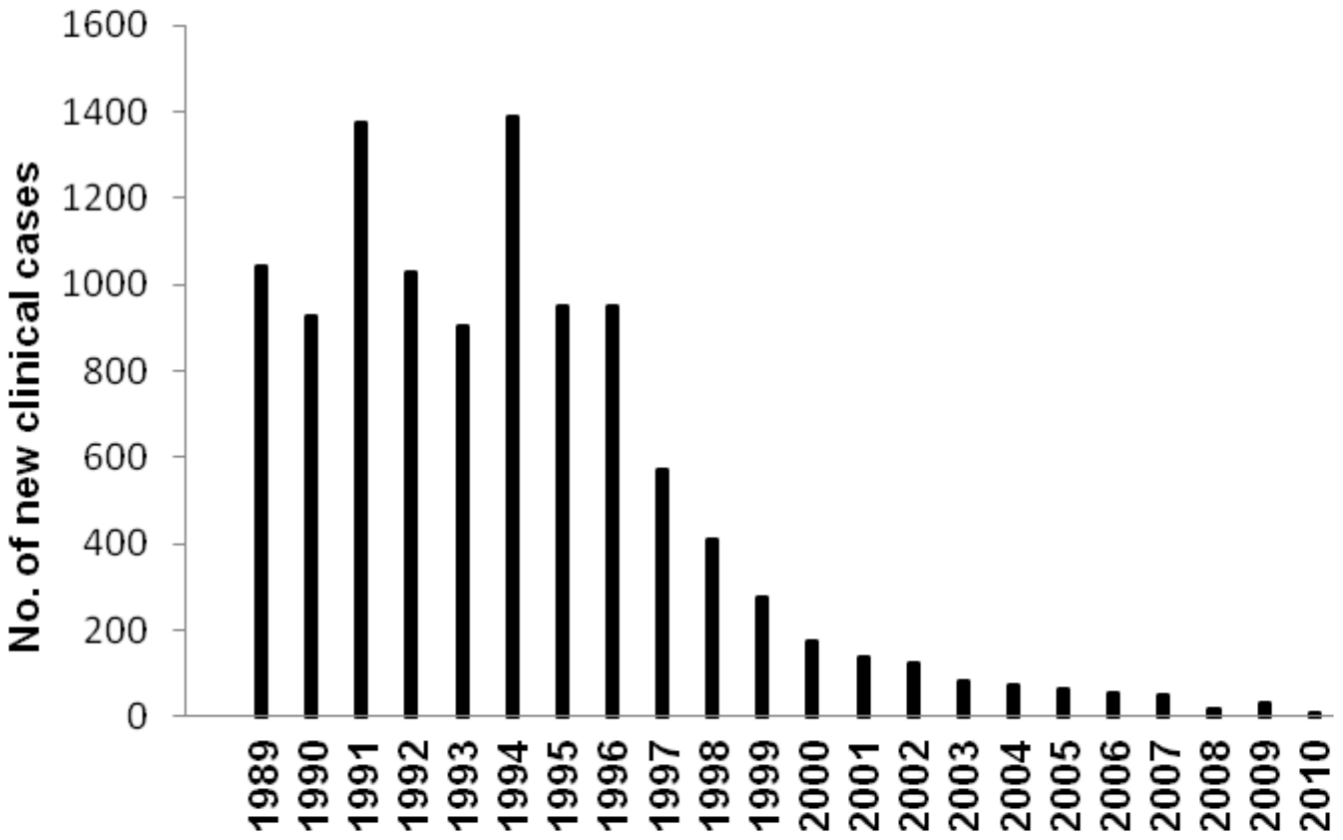
Number of new clinical cases of onchocerciasis in the Southern Chiapas focus, México. The number of new clinical cases of onchocerciasis (individuals diagnosed positive to Mazzotti reaction, nodules, or skin mf for the first time) in the Southern Chiapas focus, from 1989 to 2010.

A steady decrease of the number of individuals harboring nodules in the Southern Chiapas focus was recorded in the 1995–2010 period (Figure 3). There was also a dramatic variation in the number of new clinical cases (i.e., individuals diagnosed positive for nodules or skin microfilariae for the first time) from 1,041 individuals identified with nodules or skin microfilariae in 1989 to just 9 such individuals in 2010 (Figure 4).

## Discussion

The epidemiological and entomological parameters presented in this study strongly suggest that transmission of *O. volvulus* has been interrupted in the Southern Chiapas focus. Of the communities examined in this paper, no entomological evidence for ongoing transmission was detectable when the studies reported were completed in 2011. In Las Golondrinas and José María Morelos, where pre-control entomological data were available, Mectizan treatment has reduced transmission by greater than 99% when compared with the seasonal transmission potential of about 20 L3s per person per year that existed prior to initiating Mectizan treatment [5], [7], [19]. This meets the criterion proposed by WHO for a “near absence” of transmission for areas where pre-treatment data on transmission exist.

Given that pre-treatment data for the level of transmission do not exist for most other communities within this focus, it is not possible to quantify the effect of Mectizan on transmission in most foci as is possible for Las Golondrinas. However, as mentioned in the introduction, OEPA has set a threshold of less than one infective fly per 2,000 flies tested as the current criterion for interruption of transmission [2], [3], [4]. In 2008, the upper limit of the 95% confidence interval of the infective rate varied from 0.3 to 0.5 infective flies/2,000 examined in the sentinel and extra-sentinel communities, which is well below OEPA's criterion. This situation remained unchanged in the four communities (two of which are sentinels) that were re-tested in 2011 (on average = 0.06 infective flies/2,000 tested), indicating that transmission remained interrupted in this focus.

In addition to the 1/2,000 infective fly threshold, OEPA recommends the use of Annual transmission potential (which in the present situation is equivalent to STP) to assess the status of onchocerciasis transmission, because these metrics take into account the biting rate and the prevalence of infective flies. Estimates of the ATP necessary to maintain the parasite population (the transmission breakpoint) range from 5 to 54 L3/person/year using mathematical modeling [20] and from 7.6 to 18 L3/person/year using field observations [2], [21]. All point estimates of STP were zero, and the 95%-ULCI of potential STPs in all of the communities examined were at or below the estimated transmission breakpoint (2.3–18.4, Table 2). This suggests that if conditions remain unchanged, the parasite population is likely to be on the path to local extinction.

In 2010, a detailed assessment of the prevalence of *O. volvulus* antibodies in children in the Southern Chiapas focus was conducted, assessing over 40% of the population of children of age 5 and under who were residents of the afflicted communities. Only two children harbored Ov16 antibodies from 4,230 blood spot samples examined leading to a point estimate of exposure of 0.05% and a one-sided 95% confidence interval on the estimate of the prevalence of exposure of 0.08%, which was below the cutoff established by OEPA of 0.1%.

Additional data suggest that that clinical onchocerciasis has also been eliminated in the Southern Chiapas focus. A total of 1,719 individuals were identified with nodules in this focus in 1995; this declined to 82 individuals in 2010 (Figure 3). This decline in nodule rates was mirrored in the near absence of new clinically defined cases of onchocerciasis from reports of the local health officials in the Southern Chiapas focus during the last three years. Only nine new clinical cases (i.e., individuals diagnosed positive for nodules or skin microfilariae for the first time) were reported in 2010 (Figure 4). This finding suggests that endemic onchocerciasis no longer represents a serious health risk to the endemic community in Southern Chiapas focus.

## Supporting Information

Checklist S1 STROBE Checklist.
(DOCX)
